# Collateral-pathway–oriented clinico-angiographic framework for inferior epigastric artery–related hemorrhage: embolization planning and clinical outcomes

**DOI:** 10.1186/s42155-026-00742-3

**Published:** 2026-07-29

**Authors:** Hiroyuki Tokue, Masashi Ebara, Azusa Tokue, Yoshito Tsushima

**Affiliations:** Department of Diagnostic and Interventional Radiology, Gunma University Hospital, 3-39-22 Showa-Machi, Maebashi, Gunma 371-8511 Japan

**Keywords:** Inferior epigastric artery, Transcatheter arterial embolization, Pelvic fracture, Postpartum hemorrhage

## Abstract

**Background/objectives:**

Although inferior epigastric artery (IEA)-related hemorrhage can occur in diverse settings, outcomes following transcatheter arterial embolization (TAE) vary according to collateral anatomy and physiologic severity. We aimed to describe a collateral-pathway–oriented clinico-angiographic framework for IEA-related hemorrhage and evaluate technical and clinical outcomes of TAE across these categories. Predictors of the composite clinical failure endpoint and a simple exploratory descriptive score were assessed as exploratory secondary analyses.

**Methods:**

We retrospectively reviewed 62 consecutive patients with IEA-related hemorrhagic events treated with TAE; cases were categorized into four collateral-pathway–oriented clinico-angiographic categories (Types A–D) based on CT/DSA findings together with the clinical context, to reflect the dominant collateral circuit relevant to embolization planning. The categories were Type A (isolated abdominal wall, *n* = 28), Type B (round ligament/postpartum, *n* = 15), Type C (corona mortis/pelvic fracture, *n* = 16), and Type D (iatrogenic puncture-related, *n* = 3). Technical success was defined as angiographic disappearance of the extravasation/pseudoaneurysm. Clinical success was defined as hemodynamic stabilization within 24 h without repeat TAE or surgery. Clinical failure comprised lack of clinical success and/or in-hospital death. Rebleeding from the treated IEA territory was also assessed separately. Logistic regression was performed after excluding Type D patients (*n* = 59) owing to complete separation as an exploratory analysis of factors associated with the composite clinical failure endpoint. A point-based score was derived descriptively from these factors.

**Results:**

Technical success was achieved in all cases (62/62, 100%). The overall clinical success rate was 82.3% (51/62). Clinical success rates varied across categories: Type A 96.4%; Type B, 80.0%; Type C, 56.3%; and Type D, 100%. Rebleeding from the treated IEA territory occurred in 3 patients (4.8%), all of whom had Type C. In-hospital mortality was 12.9% (8/62), concentrated in Type C. In exploratory multivariable analysis (*n* = 59), Type C category (aOR, 4.6), DIC (aOR, 3.7), and SBP < 80 mmHg (aOR, 3.1) were associated with the composite clinical failure endpoint. A descriptive three-factor score (Type C = 2, DIC = 2, and SBP < 80 mmHg = 1) showed a stepwise increase in this composite endpoint from 7.1 to 71.4%.

**Conclusions:**

A collateral-pathway–oriented clinico-angiographic framework may support systematic evaluation of the external iliac/IEA system during embolization planning. Poorer outcomes in pelvic fracture–related Type C cases should be interpreted cautiously because they may reflect trauma burden, coagulopathy, and systemic derangement rather than IEA embolization failure alone.

**Supplementary Information:**

The online version contains supplementary material available at 10.1186/s42155-026-00742-3.

## Introduction

Inferior epigastric artery (IEA) injury is an underrecognized cause of major hemorrhage in several clinical settings. The IEA arises from the external iliac artery and ascends within the rectus sheath to anastomose with the superior epigastric artery and gives rise to pubic branches caudally that anastomose with the obturator artery to form an arterial corona mortis along the superior pubic ramus [1, 2]. In women, the IEA also supplies the round ligament artery, which contributes to the dense collateral network around the uterus and pelvic venous plexus [3]. Through these anastomoses, the IEA can serve as an important source of bleeding not only in spontaneous or anticoagulation-related abdominal wall hematoma, but also in postpartum hemorrhage, pelvic fracture–related hemorrhage, and iatrogenic complications following procedures such as catheter ablation or paracentesis [3–6].

Over the past two decades, several studies have described transcatheter arterial embolization (TAE) for IEA-related abdominal wall hematoma, reporting high technical and clinical success rates even in elderly or anticoagulated patients [3–6]. In contrast, postpartum hemorrhage and pelvic trauma outcomes are more heterogeneous because the IEA often functions as part of a broader collateral circuit via the round ligament artery and corona mortis. IEA embolization alone may be insufficient in patients with disseminated intravascular coagulation (DIC), multiple trauma, or placenta accreta spectrum [7–11]. Most prior reports have been framed based on the clinical syndrome—“rectus sheath hematoma,” “postpartum hemorrhage,” or “pelvic fracture”—rather than by vascular anatomy and hemodynamics [3–11]. To the best of our knowledge, IEA-related hemorrhages have rarely been classified across indications according to clinico-angiographic categories, and TAE outcomes have not been systematically compared among such categories. Furthermore, predictors of “clinical failure” despite technically successful IEA embolization remain poorly characterized.

Therefore, the aims of this single-center study were (1) to describe a collateral-pathway–oriented clinico-angiographic framework for IEA-related hemorrhage based on CT/DSA findings and relevant clinical context and (2) to compare technical and clinical outcomes of TAE across these categories. As an exploratory secondary analysis, we evaluated factors associated with the composite clinical failure endpoint and derived a simple descriptive score to summarize overall adverse clinical trajectory after IEA-related hemorrhage.

## Materials and methods

### Study design and ethics

This was a single-center retrospective observational study of patients who underwent TAE for IEA-related hemorrhage. The study protocol was approved by the Institutional Review Board (IRB) of Gunma University Hospital (approval no. HS2025-337; approval date: February 24, 2026), and complied with the Declaration of Helsinki and the Strengthening the Reporting of Observational Studies in Epidemiology statement. The requirement for written informed consent was waived, and an opt-out procedure was implemented, as approved by the IRB.

### Patients

We reviewed all patients who underwent TAE targeting the IEA or its branches at our institution between January 2011 and December 2024. Eligible cases met the following criteria:

1. Contrast-enhanced CT demonstrating active contrast extravasation, pseudoaneurysm, or hematoma within the anterior abdominal wall or pelvic region in a distribution compatible with the course of the IEA or its branches.
2. DSA confirmed active bleeding from the IEA or its branches (contrast extravasation or pseudoaneurysms).
3. Performance of TAE with the specific intention of occluding IEA and/or IEA-derived branches responsible for hemorrhage.

When more than one TAE session was performed for the same bleeding event, the procedure was considered a single event. After applying these criteria, 62 distinct IEA-related bleeding events in 62 patients were included in the final analyses.

#### Contrast-enhanced CT protocol

Contrast-enhanced CT was performed using multidetector CT scanners available during the study period to detect active extravasation or pseudoaneurysm in the abdominal wall or pelvis before angiography. In most patients, a standard institutional emergency protocol was used: non-contrast CT followed by contrast-enhanced imaging in the arterial and portal venous phases. Iodinated contrast material (300–370 mg I/mL) was administered intravenously at 80–100 mL with an injection rate of 3–4 mL/s, followed by a 30–40-mL saline flush. Scan timing was determined using bolus tracking with an ROI in the abdominal aorta and a threshold of approximately 150 HU (arterial phase trigger), and the portal venous phase was acquired using a fixed delay of 60–70 s after contrast initiation. Acquisition was performed with thin collimation (approximately 0.5–1.25 mm) and images were reconstructed at 0.5–1.0-mm slice thickness with multiplanar reformations as needed. When outside-hospital CT studies were used, only examinations adequate for assessing (i) extravasation/pseudoaneurysm and (ii) hemorrhage distribution relevant to the clinico-angiographic classification were included.

Across the study period, the institutional purpose and workflow of contrast-enhanced CT for acute hemorrhage (to identify active extravasation/pseudoaneurysm and hematoma distribution to guide angiography) remained essentially unchanged, and all examinations were required to be adequate for these determinations.

### Collateral-pathway–oriented clinico-angiographic framework for IEA-related hemorrhage

Based on contrast-enhanced CT, DSA findings, and relevant clinical context, IEA-related hemorrhage was categorized into four collateral-pathway–oriented clinico-angiographic categories reflecting the dominant IEA-related collateral pathway relevant to embolization planning.

#### Rationale for classification

Although the bleeding etiology (e.g., postpartum, trauma, iatrogenic puncture) is often clinically apparent before angiography, our classification was designed to be *collateral-pathway oriented* rather than purely etiologic. Specifically, it provides a structured way to (i) anticipate the dominant collateral circuit (rectus-sheath branches, IEA–round ligament pathway, corona mortis/pubic branches, or puncture-site injury), (ii) guide systematic angiographic search for “backdoor” inflow from the external iliac system, and (iii) define practical embolization targets/endpoints when bleeding persists or recurs despite treatment of other pelvic territories. Type B is intentionally defined as a pregnancy-specific phenotype in which the IEA–round ligament pathway becomes clinically relevant; thus, the purpose of the Type B label is not to isolate an anatomy-only effect from pregnancy physiology, but to standardize when and how the external iliac/IEA system (including the round ligament artery when present) should be interrogated as a potential “backdoor” source during postpartum hemorrhage management.

We emphasize that the framework is not intended to attribute outcomes to the “category itself” as a causal factor. Rather, it operationalizes the dominant IEA-related collateral pathway that must be evaluated and, when necessary, disconnected during embolization planning. Because clinical severity differs across settings (e.g., polytrauma vs postpartum hemorrhage), we performed sensitivity analyses using clinical etiology/setting in place of the clinico-angiographic category to assess robustness of associations (Supplementary Table S3a).

- Type A: isolated abdominal wall pattern

**Fig. 1:**
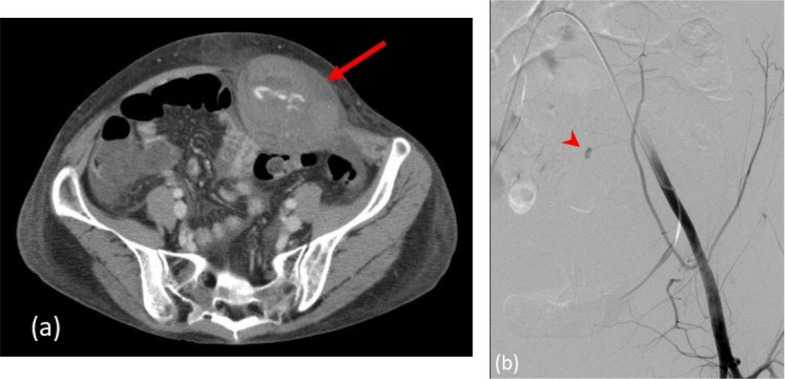
Spontaneous rectus sheath hematoma in a 37-year-old woman with cirrhosis. **a** Contrast-enhanced computed tomography demonstrated an abdominal wall hematoma with active contrast extravasation (arrow). **b** Subsequent angiography shows contrast extravasation from a branch of the left inferior epigastric artery (arrowhead). Transcatheter arterial embolization was performed using coils

Hematoma confined to the rectus sheath or adjacent abdominal wall without associated pelvic fracture or uterine/pelvic hematoma. Angiography revealed contrast extravasation or a pseudoaneurysm arising from the IEA trunk or its abdominal wall branches without prominent opacification of the round ligament artery or corona mortis (Fig. 1).

Type A was defined as abdominal wall hemorrhage without a clear temporal/spatial relationship to a recent puncture or access tract.

- Type B: round-ligament pattern (postpartum pattern)

**Fig. 2:**
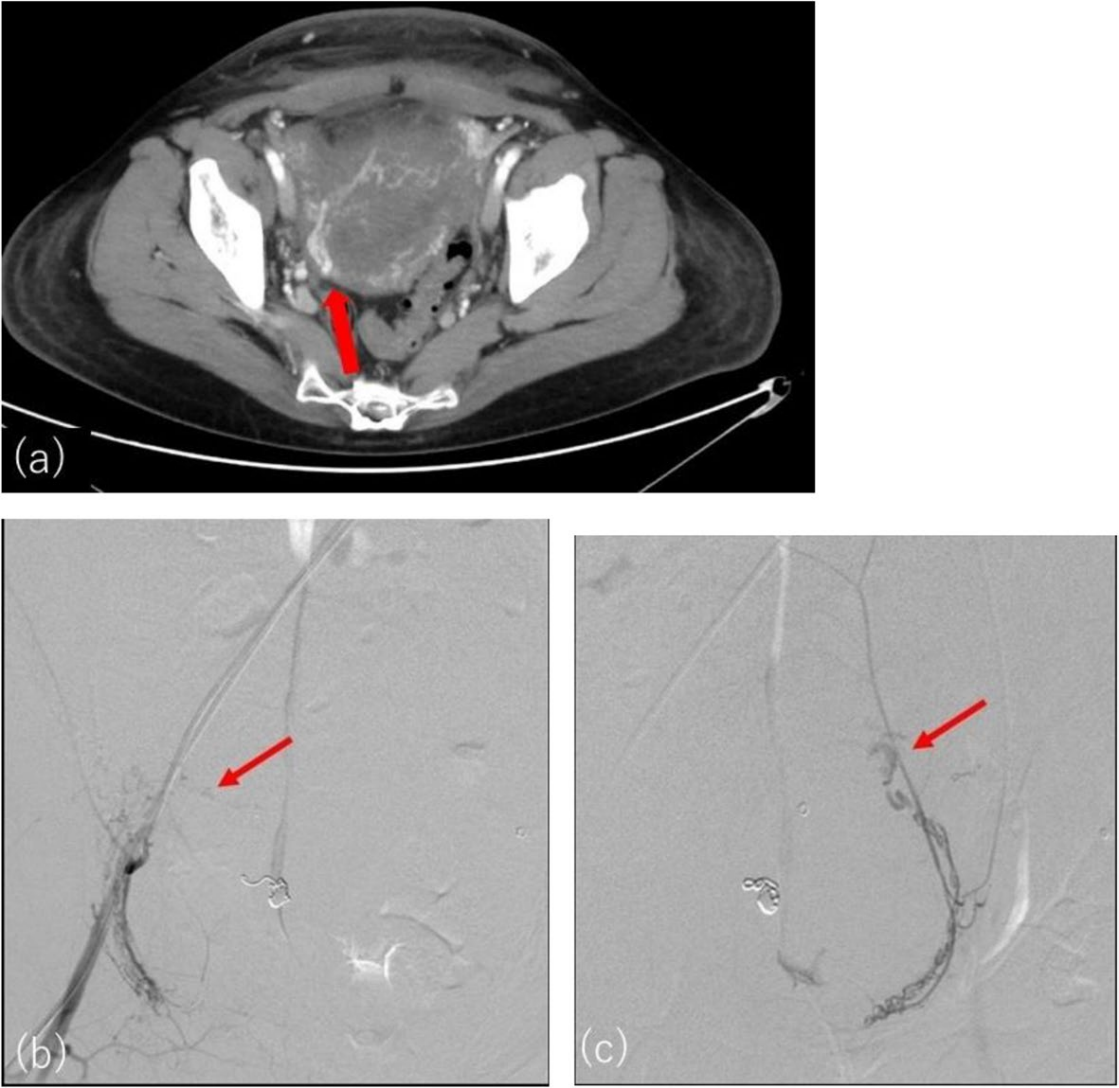
Severe postpartum hemorrhage due to retained placenta in a 31-year-old woman. Contrast-enhanced computed tomography demonstrated periuterine hematoma near the round ligament with active extravasation (arrow) (**a**). Bilateral uterine arteries were embolized with gelatin sponge and coils; however, hemodynamic instability persisted. **b**, **c** Angiography demonstrated active contrast extravasation from the bilateral round ligament arteries arising from the inferior epigastric arteries (arrows). Additional transcatheter arterial embolization was performed using gelatin sponge and coils

Hematoma and/or active contrast extravasation around the uterine body and round ligament in the setting of postpartum hemorrhage without pelvic fracture. DSA revealed the round ligament artery arising from the IEA with distal extravasation around the uterus. In some cases, IEA/round ligament bleeding was observed as a residual or recurrent hemorrhage following prior uterine and/or internal iliac artery embolization [3, 8, 10] (Fig. 2).

- Type C: corona mortis/pelvic-fracture pattern

**Fig. 3:**
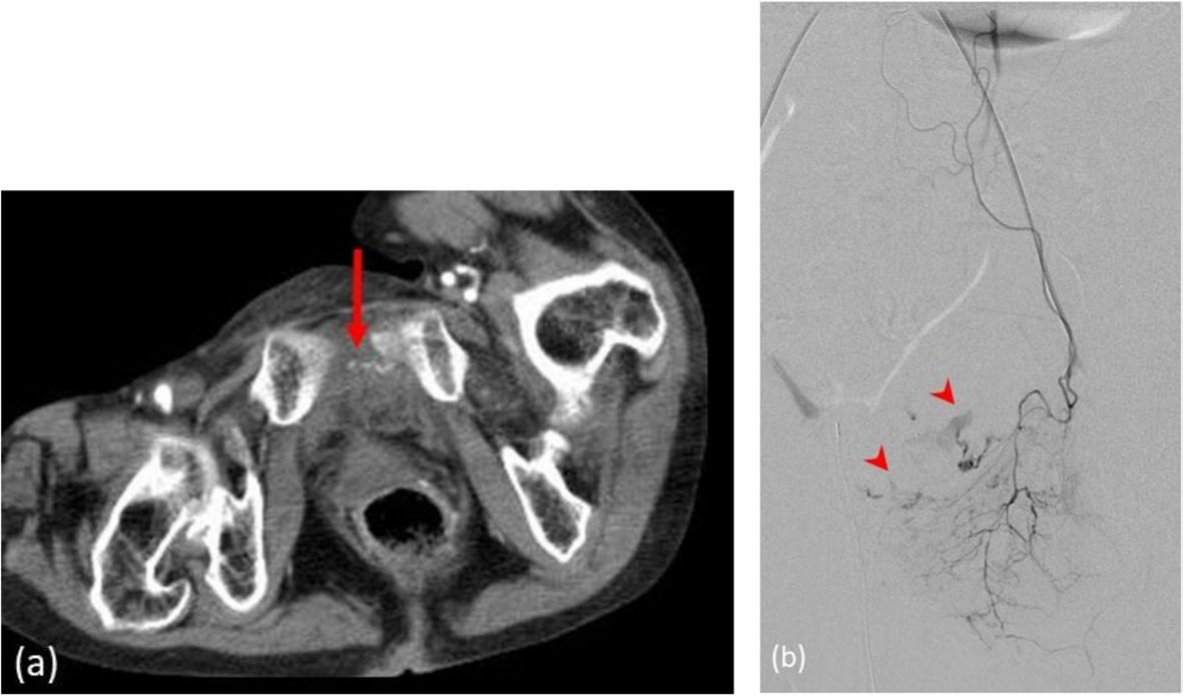
A 72-year-old woman presented with hemodynamic instability with multiple injuries after a traffic accident. **a** Contrast-enhanced computed tomography demonstrated active contrast extravasation adjacent to a left pubic ramus fracture (arrow). **b** Angiography revealed extravasation from a pubic branch supplied via an anastomosis between a descending branch of the left inferior epigastric artery and the pubic branch (arrowheads). Transcatheter arterial embolization was performed using gelatin sponge

Hematoma and/or contrast extravasation adjacent to pubic rami fractures or other pelvic fractures. Angiography revealed extravasation from the pubic branches of the IEA, the arterial corona mortis, or IEA-derived inflow into the obturator territory [5, 9] (Fig. 3).

- Type D: iatrogenic puncture-related pattern

**Fig. 4:**
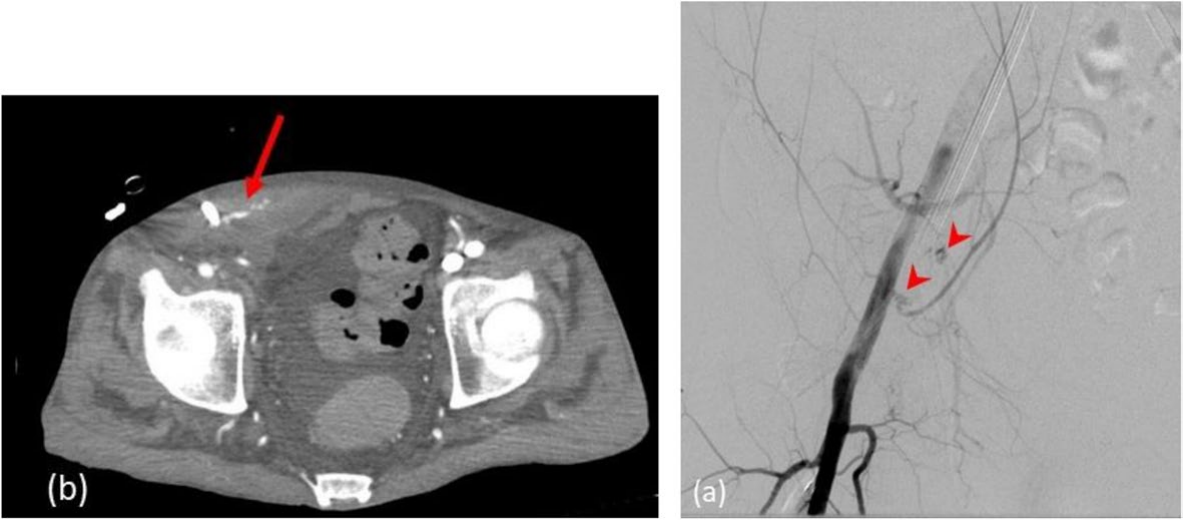
Hematoma in a 45-year-old man. A 12-Fr temporary hemodialysis catheter was placed in the right femoral vein for management of chronic kidney disease, after which hematoma developed in the groin and anterior abdominal wall. **a** Contrast-enhanced computed tomography suggested injury to the right inferior epigastric artery (arrow). **b** Angiography confirmed injury to the right inferior epigastric artery with active extravasation from an inferior epigastric artery branch (arrowheads). Transcatheter arterial embolization was performed with N-butyl-2-cyanoacrylate

Hematoma with active extravasation in the abdominal wall near iatrogenic puncture sites, such as paracentesis, abdominal drains, or common femoral artery access. DSA revealed injury to the IEA or its superficial branches with distal extravasation corresponding to the puncture site [12, 13] (Fig. 4). Type D required a *recent puncture-related clinical context* and angiographic concordance between the bleeding point and the puncture/access/drain tract (i.e., direct IEA trunk/superficial branch injury near the tract).

Two interventional radiologists with expertise in emergency and obstetric/trauma embolization independently reviewed the CT and DSA images and assigned the clinico-angiographic category. Discrepancies were resolved by consensus.

**Fig. 5:**
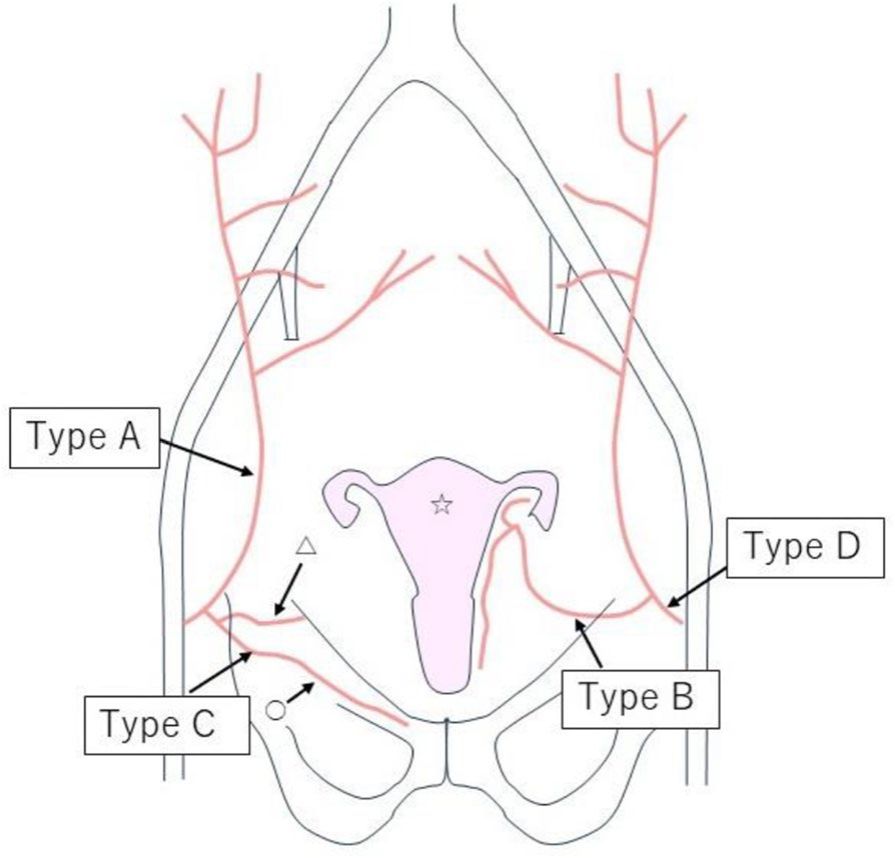
Schematic illustration of the inferior epigastric artery and relevant collateral pathways for embolization planning. The diagram summarizes the collateral-pathway–oriented clinico-angiographic framework for IEA-related hemorrhage. Type A represents isolated abdominal wall or rectus sheath branch bleeding. Type B represents the IEA–round ligament pathway contributing to periuterine bleeding in postpartum hemorrhage. Type C represents pubic branch/corona mortis involvement in pelvic fracture–related hemorrhage, including potential collateral communication with the obturator territory. Type D represents puncture-tract injury involving the IEA or its superficial branches. This schematic is intended to support systematic interrogation of the external iliac/IEA system during embolization planning, rather than to imply that the categories represent etiology-independent prognostic factors. Symbols indicate the uterus (☆), pubic branch (○), and obturator branch (△)

A schematic illustration of the IEA and its relevant collateral pathways is provided in Fig. 5 to clarify the anatomical basis of the collateral-pathway–oriented clinico-angiographic framework. The diagram highlights the rectus sheath branches, the IEA–round ligament pathway, the pubic branch/corona mortis connection, and puncture-related IEA injury as operational targets for embolization planning.

### Embolization technique

All procedures were performed using a common femoral artery approach under local anesthesia and conscious sedation when indicated. A 4- or 5-Fr catheter was positioned in the external iliac artery, and selective angiography was performed to identify the IEA and its branches. The coaxial microcatheter system was advanced into the culprit branch as selectively as possible.

Selective angiography of the external iliac artery and IEA was performed using a 4–5 Fr diagnostic catheter. Superselective catheterization was achieved with a coaxial microcatheter system (1.7–2.0 Fr microcatheter over a 0.014–0.016-inch guidewire) advanced into the culprit IEA branch as distally as feasible.

After confirming the bleeding point on superselective angiography, the embolic material was selected according to the clinico-angiographic category, bleeding severity, and coagulation status. The following agents were used alone or in combination:

- Gelatin sponge (GS) particles
- Detachable or pushable metallic coils
- N-butyl-2-cyanoacrylate (NBCA) mixed with ethiodized oil

Embolic agents included GS particles, detachable or pushable coils, and NBCA mixed with ethiodized oil. Coils were deployed to achieve focal exclusion of pseudoaneurysms or to secure a distal/superselective position when a discrete bleeding point was identified. GS was used for diffuse oozing or small-vessel injury when superselective delivery was possible. NBCA was preferentially used in patients with severe coagulopathy and/or profound hemodynamic instability requiring rapid, coagulation-independent hemostasis; NBCA was prepared by mixing with ethiodized oil at an operator-selected ratio (1:2–1:4, adjusted to flow and target level), and the microcatheter was flushed with 5% dextrose solution before injection to prevent premature polymerization.

The procedural endpoint was defined as complete angiographic disappearance of contrast extravasation and/or pseudoaneurysm opacification on final control angiography of the target vessel. When extensive collateralization was suspected (Types B/C), embolization was extended as needed to disconnect the culprit branch from potential collateral backflow while avoiding non-target embolization.

In Type A cases, the default strategy was superselective GS embolization of the responsible branch with adjunctive coils for well-defined pseudoaneurysms. In hemodynamically unstable patients with DIC, NBCA alone was employed to rapidly occlude the IEA trunk and/or bleeding branch, particularly in the setting of coagulopathy or hemodynamic instability, where immediate and durable hemostasis is required.

In Type B and C cases, embolization of the uterine and/or internal iliac arterial territories was performed as needed, followed by additional IEA embolization when persistent or recurrent IEA-related bleeding was observed. For Type D cases, the goal was complete exclusion of the injured segment with coils and/or GS while preserving the uninvolved branches whenever feasible. Embolization targeted the IEA and/or its culprit branches, including the round ligament artery, when it arose from the IEA, based on CT/DSA findings. Embolization was performed on the ipsilateral or bilateral IEA, as indicated by the CT/DSA findings; laterality was recorded but not used for category classification.

All procedures were performed by or under the direct supervision of board-certified interventional radiologists. Periprocedural management, including transfusion, resuscitation, and correction of coagulopathy, followed the institutional trauma and obstetric hemorrhage protocols.

### Data collection and endpoint definitions

Clinical and imaging data were extracted from electronic medical records and a picture archiving and communication system. The following variables were recorded.

- Baseline characteristics: age, sex, and underlying conditions (e.g., liver cirrhosis, malignancy, pregnancy/delivery, and trauma).
- Physiological status at TAE: systolic blood pressure (SBP), heart rate, shock index, serum lactate level, and base excess.
- Coagulation status: Presence of DIC according to the Japanese Ministry of Health, Labour and Welfare criteria, prothrombin time–international normalized ratio (PT-INR), and platelet count.
- Bleeding-related indices: red blood cell (RBC) transfusion volume within 24 h before TAE.
- Clinico-angiographic category: Type A–D as defined above.
- Procedural details: embolic materials, need for adjunctive uterine or internal iliac artery embolization, and procedure time.

Outcomes were defined as follows:

- Technical success: Successful catheterization of the culprit vessel(s) with complete angiographic disappearance of contrast extravasation or pseudoaneurysm at the end of the procedure.
- Clinical success: Hemodynamic stabilization within 24 h after TAE without the need for repeat TAE or surgical hemostasis for the same bleeding event.
- Clinical failure: Composite endpoint of lack of clinical success (persistent or recurrent bleeding requiring repeat TAE or surgery) and/or in-hospital death from any cause.
- Rebleeding: Renewed hemorrhage from the same IEA territory documented on CT and/or angiography after an initially successful TAE.
- In-hospital mortality: Death from any cause during the index hospitalization.

*Post-procedural follow-up*: After TAE, patients were managed per institutional emergency/obstetric/trauma pathways with serial reassessment of vital signs and bleeding-related laboratory tests (e.g., hemoglobin/hematocrit and coagulation parameters). Post-TAE transfusion requirements were recorded. Follow-up imaging (contrast-enhanced CT, or repeat angiography) was performed only when clinically indicated for persistent instability or suspected recurrent bleeding.

For sensitivity analyses, we evaluated (i) IEA-territory rebleeding alone and (ii) a hemostasis-focused endpoint defined as IEA-territory rebleeding and/or the need for additional hemostatic intervention, excluding death-only cases without documented IEA-territory rebleeding or additional hemostatic intervention.

Because clinical decision-making in pelvic trauma is outcome-driven, clinical failure was defined as a pragmatic composite endpoint (need for further hemostatic management and/or in-hospital death), while IEA-territory rebleeding was separately recorded as a vessel-specific hemostatic outcome.

Missing data were handled as follows. For each variable, completeness was assessed at the time of database extraction. Variables defining clinico-angiographic category and the primary outcomes (technical success, clinical success/failure, IEA-territory rebleeding, and in-hospital mortality) were available for all included cases. For regression analyses, we performed complete-case analysis (listwise deletion), such that patients with missing values in any candidate predictor were excluded from the corresponding model. Because the number of missing values was small and the study sample was limited, no imputation was performed. The denominator for each analysis is therefore reported explicitly (e.g., regression analysis set: *n* = 59 after excluding Type D).

### Statistical analysis

Continuous variables are presented as medians with interquartile ranges (IQRs), and categorical variables as counts and percentages. Group comparisons across clinico-angiographic categories (types A–D) were performed using the Kruskal–Wallis test for continuous variables and the chi-squared test or Fisher’s exact test for categorical variables, as appropriate.

To explore factors associated with the composite clinical failure endpoint, we first performed univariable logistic regression using clinically plausible candidate variables informed by the trauma and postpartum hemorrhage literature: age, sex, clinico-angiographic category (Type A as the reference), presence of disseminated intravascular coagulation (DIC), systolic blood pressure (SBP) < 80 mmHg before TAE, serum lactate level, RBC transfusion ≥ 10 units within 24 h before TAE, and presence of multiple trauma. Variables showing at least one suggestive association in the univariable analysis (*p* < 0.10) were considered for multivariable modeling. Given the limited number of clinical failure events, the final multivariable model was restricted to a small set of nonredundant predictors to preserve model stability and avoid overfitting. Collinearity among predictors was assessed; variables reflecting advanced shock and bleeding burden (e.g., lactate and RBC transfusion) were not entered into the final model owing to collinearity with SBP and DIC, and multiple trauma was not included because it occurred exclusively within the pelvic fracture–related Type C category in this cohort. Adjusted odds ratios (aORs) with 95% confidence intervals (CIs) are reported. Statistical significance was defined as a two-sided *p*-value < 0.05.

Sex was evaluated in univariable analysis; however, because Type B occurred exclusively in female patients, sex was not entered into the multivariable model due to complete confounding/collinearity with the Type B category.

As an exploratory secondary analysis, a simple descriptive score was constructed from the factors retained in the final model by assigning integer points approximately proportional to the regression coefficients. This score was intended to summarize overall adverse clinical trajectory after IEA-related hemorrhage, rather than to represent vessel-specific failure of IEA embolization.

## Results

### Patient characteristics and clinico-angiographic categories

A total of 62 consecutive IEA–related hemorrhagic events treated with TAE were included. The cases were classified into four predefined collateral-pathway–oriented clinico-angiographic categories: Type A (isolated abdominal wall, *n* = 28), Type B (round ligament/postpartum, *n* = 15), Type C (corona mortis/pelvic fracture, *n* = 16), and Type D (iatrogenic puncture-related, *n* = 3) (Table 1). DIC was present in 26 patients (41.9%) and profound hypotension (SBP < 80 mmHg) was observed in 22 patients (35.5%) (Table 1). Polytrauma was present in 10 patients (16.1%), all of whom belonged to Type C. All Type C cases included unstable pelvic ring injuries, with polytrauma in 10 of 16 cases (Table 1). DIC and profound hypotension (SBP < 80 mmHg) were more frequent in Types B and C than in Type A (DIC: Type A 6/28 [21.4%], Type B 9/15 [60.0%], Type C 10/16 [62.5%], Type D 1/3 [33.3%]; SBP < 80 mmHg: Type A 5/28 [17.9%], Type B 7/15 [46.7%], Type C 9/16 [56.3%], Type D 1/3 [33.3%]).

**Table 1:** Baseline characteristics of 62 patients with IEA-related hemorrhage managed by TAE

Characteristic	All patients (*n* = 62)
Age, years, median (IQR)	62 (48–74)
Male sex, *n* (%)	32 (51.6)
**Clinico-angiographic category/clinical context, *n* (%)**	
Type A: isolated abdominal wall	28 (45.2)
– Spontaneous	25
– Postoperative	3
Type B: round-ligament/postpartum	15 (24.2)
– Retained placenta	4
– Uterine atony	11
Type C: corona mortis/pelvic-fracture	16 (25.8)
– Unstable pelvic ring injury	16
– With polytrauma (subset of Type C)	10
Type D: iatrogenic puncture-related (femoral access or drain site)	3 (4.8)
**Physiologic/clinical severity markers, *n* (%)**	
DIC present	26 (41.9)
SBP < 80 mmHg before TAE	22 (35.5)
Polytrauma present (entire cohort)	10 (16.1)

Denominators reflect available data; no missing values were present for clinico-angiographic category assignment

*Abbreviations*: *IEA* inferior epigastric artery, *TAE* transcatheter arterial embolization, *IQR* interquartile range, *DIC* disseminated intravascular coagulation, *SBP* systolic blood pressure

### Embolization strategies and technical performance

Embolization was predominantly based on GS, with the selective use of coils and NBCA, based on the angiographic and clinical context (Table 2). When embolic material categories were defined as mutually exclusive (“NBCA only” when embolization was performed with NBCA alone; otherwise “Coils ± GS” if coils were used; otherwise “GS alone”), GS alone was used in 48 cases (77.4%), Coils ± GS in 11 (17.7%), and NBCA only in 3 (4.8%) (Table 2). Adjunctive uterine and/or internal iliac artery embolization was performed only in postpartum (Type B, 2/15 [13.3%]) and pelvic fracture–related cases (Type C, 3/16 [18.8%]) (Table 2). Technical success was achieved in all procedures (62/62, 100.0%) (Table 2).

**Table 2:** Angiographic/embolization characteristics and clinical outcomes by clinico-angiographic category

Variable	All (*n* = 62)	Type A (*n* = 28)	Type B (*n* = 15)	Type C (*n* = 16)	Type D (*n* = 3)
**Side of IEA embolization: right/left/bilateral**	20/36/6	8/20/0	4/8/3	5/8/3	3/0/0
**Embolic material, *n* (%)**					
GS alone	48 (77.4)	26 (92.9)	11 (73.3)	10 (62.5)	1 (33.3)
Coils ± GS	11 (17.7)	2 (7.1)	3 (20.0)	4 (25.0)	2 (66.7)
NBCA only	3 (4.8)	0 (0.0)	1 (6.7)	2 (12.5)	0 (0.0)
**Technical/adjunctive procedures, *n* (%)**					
Technical success	62 (100.0)	28 (100.0)	15 (100.0)	16 (100.0)	3 (100.0)
Additional uterine and/or internal iliac artery embolization	5 (8.1)	0 (0.0)	2 (13.3)	3 (18.8)	0 (0.0)
**Clinical outcomes, *n* (%)**					
Clinical success	51 (82.3)	27 (96.4)	12 (80.0)	9 (56.3)	3 (100.0)
Clinical failure^*^	11 (17.7)	1 (3.6)	3 (20.0)	7 (43.8)	0 (0.0)
Rebleeding from same IEA territory†	3 (4.8)	0 (0.0)	0 (0.0)	3 (18.8)	0 (0.0)
In-hospital mortality	8 (12.9)	1 (3.6)	0 (0.0)	7 (43.8)	0 (0.0)

^*^Clinical failure was defined as the absence of clinical success (persistent or recurrent bleeding requiring repeat TAE or surgical hemostasis) and/or in-hospital death from any cause

^†^Rebleeding was defined radiologically (CT and/or angiography) as a renewed hemorrhage from the same IEA territory after the initial successful TAE

All outcome variables shown in this table were available for all patients

*Abbreviations*: *IEA* inferior epigastric artery, *TAE* transcatheter arterial embolization, *GS* gelatin sponge, *NBCA* n-butyl-2-cyanoacrylate

### Clinical outcomes according to clinico-angiographic category

Overall, clinical success was achieved in 51 patients (82.3%), whereas clinical failure occurred in 11 (17.7%) (Table 2). Clinical success rates varied across the clinico-angiographic categories, being highest in types A (27/28, 96.4%) and D (3/3, 100.0%), intermediate in Type B (12/15, 80.0%), and lowest in Type C (9/16, 56.3%) (Table 2). Rebleeding from the same IEA territory occurred in three patients (4.8%), all within Type C (3/16, 18.8%) (Table 2). The in-hospital mortality was 12.9% (8/62), occurring in types A (1/28, 3.6%) and C (7/16, 43.8%), with no deaths in types B or D (Table 2). In Type C, angiographically confirmed IEA-territory rebleeding was documented in 3 patients (3/16), whereas the remaining clinical failures reflected the composite endpoint (death and/or need for additional hemostatic management) without documented IEA-territory rebleeding. In Type C patients, additional major pelvic arterial bleeding requiring embolization beyond the IEA/corona mortis target was identified in 3 of 16 patients (18.8%): the internal pudendal artery (*n* = 1) and lateral sacral artery (*n* = 2). The remaining Type C patients had angiographically evident IEA-derived pubic branch/corona mortis bleeding without another major embolized pelvic arterial source. However, because clinical failure was defined as a composite endpoint including all-cause in-hospital death, we additionally report IEA-territory rebleeding as a separate outcome and present hemostasis-focused sensitivity analyses that exclude death-only cases without documented IEA-territory rebleeding or additional hemostatic intervention (Supplementary Table S3b, S3c). All Type C clinical failures met the composite endpoint through in-hospital death, with or without documented IEA-territory rebleeding, supporting the possibility that systemic physiologic derangement and concomitant injuries contributed to adverse outcomes beyond recurrent bleeding from the treated IEA territory.

### Exploratory analysis of factors associated with the composite clinical failure endpoint

Regression analyses were performed after excluding Type D owing to the small sample size (*n* = 3) and zero clinical failure events (complete separation), yielding an analysis set of *n* = 59 (Types A–C). In univariable logistic regression (*n* = 59), Type C category, DIC, SBP < 80 mmHg, lactate ≥ 4 mmol/L, RBC transfusion ≥ 10 units within 24 h before TAE, and multiple trauma showed suggestive associations with clinical failure (Table 3). In univariable analysis, sex showed separation with Type B (postpartum) because all Type B cases were female; therefore, sex was not included in multivariable modeling to avoid redundancy and unstable estimates. For multivariable modeling, lactate and RBC transfusions were excluded owing to collinearity with SBP and DIC, and multiple trauma was excluded because it occurred exclusively within the Type C group (complete confounding). In the final multivariable model (*n* = 59), Type C category (aOR 4.6; 95% CI 1.3–16.2; *p* = 0.02), DIC (aOR 3.7; 95% CI 1.1–12.3; *p* = 0.03), and SBP < 80 mmHg (aOR 3.1; 95% CI 1.0–9.7; *p* = 0.047) were independently associated with the composite clinical failure endpoint in this exploratory model (Table 3). Based on these factors, a simple exploratory descriptive score (Type C, 2 points; DIC, 2 points; SBP < 80 mmHg, 1 point) demonstrated stepwise increase in the observed clinical failure: 7.1% (3/42) for scores 0–1, 23.1% (3/13) for scores 2–3, and 71.4% (5/7) for scores 4–5 (Table 4). In Firth’s penalized likelihood regression, the direction and clinical interpretation of the associations were unchanged, with more conservative effect estimates (Table 3).

**Table 3:** Exploratory logistic regression analysis of factors associated with the composite clinical failure endpoint after IEA-related TAE. (Regression analysis set: Type D excluded (*n* = 59). Complete-case analysis was used for regression (no imputation)

**(A) Univariable analysis (*n*** **= 59)**			
**Variable**	**Univariable OR (95% CI)**	***p*** **value**	
Type C vs. Type A	18.2 (4.0–81.9)	< 0.001	
Type B vs. Type A	5.1 (0.9–29.4)	0.07	
DIC present	6.0 (1.8–19.6)	0.003	
SBP < 80 mmHg before TAE	5.5 (1.7–17.6)	0.004	
Lactate ≥ 4 mmol/L	4.0 (1.2–13.0)	0.02	
RBC transfusion ≥ 10 units (24 h before TAE)	3.8 (1.1–13.1)	0.03	
Multiple trauma present	3.2 (0.9–11.6)	0.08	
Male sex	NE (complete separation)	NE	
**(B) Multivariable analysis (*n*** **= 59): MLE vs Firth (bias-reduced)**			
**Variable**	**Multivariable aOR (95% CI)**	***p*** **value (MLE)**	**Firth aOR (95% CI)**
Type C vs. Type A	4.6 (1.3–16.2)	0.02	3.8 (1.1–13.2)
DIC present	3.7 (1.1–12.3)	0.03	3.1 (1.0–10.0)
SBP < 80 mmHg before TAE	3.1 (1.0–9.7)	0.047	2.6 (0.9–8.0)

Type D was excluded owing to the small sample size (*n* = 3) and absence of clinical failure events (complete separation)

Lactate ≥ 4 mmol/L and RBC transfusion ≥ 10 units were excluded from the multivariable model because of collinearity with SBP and DIC

Multiple traumas were not included in the multivariable model because they occurred exclusively in the Type C group (complete confounding)

NE indicates that the OR was not estimable due to complete or quasi-separation in the corresponding univariable model

Sex was not included in the multivariable model because Type B was restricted to postpartum women; therefore, sex/pregnancy-related factors could not be separated from the Type B subgroup effect

Firth’s penalized likelihood regression was performed as a sensitivity analysis to reduce small-sample bias and assess the robustness of the conventional maximum-likelihood estimates. Abbreviations: *OR*, odds ratio; *aOR*, adjusted odds ratio; *CI*, confidence interval; *DIC*, disseminated intravascular coagulation; *SBP*, systolic blood pressure; *RBC*, red blood cell; *TAE*, transcatheter arterial embolization; *IEA*, inferior epigastric artery

**Table 4:** Exploratory descriptive score and observed composite clinical failure after IEA-related hemorrhage treated by TAE

Exploratory descriptive score group*	Definition	*n* (%)	Clinical failures, *n* (%)
0–1 points	Total score 0–1	42 (67.7)	3 (7.1)
2–3 points	Total score 2–3	13 (21.0)	3 (23.1)
4–5 points	Total score 4–5	7 (11.3)	5 (71.4)

^*^Exploratory descriptive score components: Type C category = 2 points; DIC present = 2 points; SBP < 80 mmHg before TAE = 1 point

The score was derived from the multivariable model excluding Type D (Table 3), and the score strata are shown for the entire cohort (*n* = 62) for descriptive purposes

This exploratory score was intended to summarize overall adverse clinical trajectory and should not be interpreted as a validated predictor of vessel-specific IEA embolization failure

*Abbreviations*: *DIC* disseminated intravascular coagulation, *SBP* systolic blood pressure, *TAE* transcatheter arterial embolization, *IEA* inferior epigastric artery

### Sensitivity analyses

Because the clinico-angiographic categories incorporate clinical context by design, we assessed whether similar associations would be observed when clinical etiology/setting was used instead of the proposed categories. This sensitivity analysis yielded qualitatively similar associations (Supplementary Table S3a), indicating that the proposed categories partly reflect the underlying clinical setting and disease severity. Therefore, these results should not be interpreted as evidence that the collateral pathway itself is an etiology-independent predictor of outcome.

To address potential misclassification introduced by including all-cause in-hospital death in the composite endpoint—particularly in polytrauma—we additionally performed sensitivity analyses using (i) IEA-territory rebleeding alone and (ii) a hemostasis-focused endpoint defined as IEA-territory rebleeding and/or the need for additional hemostatic intervention, excluding death-only cases without documented IEA-territory rebleeding or additional hemostatic intervention (Supplementary Tables S3b and S3c).

## Discussion

In this single-center cohort, technical success was universal, whereas clinical outcomes varied across the collateral-pathway–oriented clinico-angiographic categories. These differences should be interpreted as reflecting both collateral-pathway anatomy and the underlying clinical context, including trauma burden, coagulopathy, and systemic physiologic derangement, rather than the category alone. This approach is intentionally “clinico-angiographic” (not purely angiographic), because minimal clinical context is required to interpret which collateral pathway is operationally relevant at the time of embolization planning. Importantly, the sensitivity analysis substituting clinical etiology/setting for the clinico-angiographic categories yielded qualitatively similar associations. This finding suggests that the proposed categories partly capture underlying etiology and disease severity, rather than representing an etiology-independent angiographic determinant of outcome. Accordingly, we interpret the framework primarily as an operational tool for embolization planning and standardized collateral-pathway assessment.

Types A and D demonstrated high clinical success, whereas Type C (corona mortis/pelvic fracture) demonstrated the lowest clinical success and the highest in-hospital mortality. Rebleeding from the treated IEA territory was uncommon and occurred only in the Type C group. In the regression analyses excluding Type D (*n* = 59), the Type C category, DIC, and profound hypotension (SBP < 80 mmHg) were associated with the composite clinical failure endpoint. The exploratory three-factor score showed a stepwise increase in this composite endpoint, but should be interpreted as summarizing overall adverse clinical trajectory rather than vessel-specific failure of IEA embolization.

The IEA arises from the external iliac artery just above the inguinal ligament, enters the rectus sheath, and supplies the anterior abdominal wall through the muscular/perforating branches. It also forms a strong anastomosis with the superior epigastric artery, creating a craniocaudal epigastric arcade that can maintain bleeding or permit retrograde filling if embolization is too limited [1].

Beyond the abdominal wall, the IEA has two clinically important pelvic connections. Pubic branches may anastomose with the obturator artery (corona mortis), linking the external iliac/IEA system to the internal iliac territories, which is highly relevant in pelvic fractures where collateral backflow can contribute to persistent hemorrhage [14]. In women, the round ligament artery may arise from the IEA and can act as a “backdoor” periuterine supply in postpartum hemorrhage, particularly after uterine/internal iliac embolization, supporting targeted embolization of the IEA system (including the round ligament artery when it arose from the IEA) when DSA shows external-iliac–system supply to periuterine extravasation [8, 15].

Previous literature reports high clinical success for embolization of abdominal wall hematomas, including in anticoagulated and elderly patients, which is consistent with the favorable outcomes in Type A [16, 17]. In contrast, postpartum hemorrhage and pelvic trauma outcomes are more variable due to complex collaterals and systemic physiological derangement; accordingly, Type B cases demonstrated intermediate outcomes, and Type C cases had the worst outcomes despite uniform technical success. Our study extends prior work by evaluating these different clinical contexts under a collateral-pathway–oriented IEA-focused framework, helping explain why “IEA embolization” performs differently across settings.

Although both Type A and Type D involve the anterior abdominal wall, we distinguished puncture-tract injuries (Type D) from non–puncture-related abdominal wall bleeding (Type A) because the typical angiographic target level and embolization endpoint (segmental exclusion vs superselective branch occlusion) differ.

Several mechanisms may underlie the observed differences across categories. Type A represents relatively compartmentalized bleeding, in which exclusion of the culprit branch is often sufficient [16, 17]. Type B likely reflects a collateralized periuterine environment where the IEA–round ligament pathway can persist as an inflow, sometimes warranting adjunctive pelvic embolization [8, 15]. However, the absence of deaths suggests that once recognized, this bleeding source is usually controllable.

Accordingly, Type B should be interpreted as a pregnancy-specific operational category that prompts systematic evaluation of the IEA–round ligament collateral pathway, rather than as evidence that the round ligament anatomy independently worsens outcomes.

The presence of concomitant non-IEA pelvic arterial bleeding in some Type C patients further supports that Type C represents a broader pelvic trauma hemorrhage context rather than isolated IEA-territory bleeding. Because clinical failure included all-cause in-hospital death, the discrepancy between the low rate of angiographically confirmed IEA-territory rebleeding (*n* = 3) and the high mortality in Type C suggests that adverse outcomes may have been influenced by global physiologic derangement and/or concomitant injuries, rather than recurrent bleeding from the embolized IEA territory alone. Importantly, the framework is intended not to “discover the etiology” but to standardize how the external iliac/IEA collateral pathways are interrogated and disconnected during embolization planning—particularly when pelvic bleeding persists after embolization of internal iliac or uterine territories. This interpretation is consistent with the associations of DIC and profound hypotension with the composite clinical failure endpoint. Clinically, IEA embolization in pelvic trauma should be viewed as a component of a broader hemostatic strategy, and the exploratory descriptive score may help summarize overall adverse clinical trajectory, but should not be interpreted as a validated predictor of vessel-specific IEA embolization failure.

This study has certain limitations inherent to its retrospective single-center design. First, the sample size, particularly for Type D, limited inference across all subgroups; accordingly, Type D was excluded from the regression analyses. Second, the composite definition of clinical failure includes in-hospital death from any cause, which improves clinical relevance, but may dilute vessel-specific inferences. Third, treatment strategies (choice of embolic material, degree of superselectivity, and use of adjunctive pelvic embolization) were not randomized and may reflect operator judgment and evolving institutional protocols over a long study period. Fourth, unmeasured confounding factors are possible, especially in trauma, where injury severity metrics and competing bleeding sources may strongly influence outcomes. Fifth, because Type B occurred exclusively in postpartum women, we could not statistically disentangle the contribution of the IEA–round ligament anatomy from sex- and pregnancy-related physiology; therefore, Type B is presented for clinical pathway standardization rather than for inference of an anatomy-only causal effect. Finally, while clinico-angiographic category assignment was performed by experienced interventional radiologists, the classification may still be subject to interobserver variability and may require external validation for reproducibility.

## Conclusions

Although technical success was achieved in all cases, clinical outcomes varied across the collateral-pathway–oriented clinico-angiographic categories and physiologic severity. Poorer outcomes in pelvic fracture–related Type C cases should not be interpreted as failure of IEA embolization alone, because they likely reflect, at least in part, severe trauma burden, concomitant bleeding sources, DIC, and systemic physiologic derangement. In this setting, IEA embolization should be considered one component of early, comprehensive hemostatic and resuscitative management rather than an isolated determinant of outcome.

## Supplementary Information

Supplementary Material 1: Table S3a. Sensitivity analysis: clinical etiology/setting substituted for the clinico-angiographic category in logistic regression for clinical failure. Notes: Sensitivity analysis: logistic regression using clinical etiology/setting in place of the clinico-angiographic category. Regression analysis set: Type D excluded (n = 59). * Lactate and RBC transfusion were excluded from the multivariable model due to collinearity with SBP and DIC. † Multiple trauma was excluded from the multivariable model because it occurred exclusively within pelvic fracture cases (perfect confounding). Table S3b, S3c. Sensitivity analyses using hemostasis-focused endpoints (IEA-territory rebleeding and non-death hemostatic failure) to address potential confounding by all-cause mortality in polytrauma. Table S3b. Outcome = IEA-territory rebleeding (CT/angiography-confirmed), Type D excluded (n = 59). Notes: Outcome events: 3/59, all in Type C (consistent with Table 2). Firth penalized logistic regression was used due to sparse events and quasi-separation. Table S3c. Outcome = Hemostasis-focused failure (IEA-territory rebleeding OR additional hemostatic intervention), excluding death-only cases without documented IEA-territory rebleeding/intervention, Type D excluded (n = 59).

## Data Availability

The data presented in this study are available from the corresponding author upon reasonable request. The data are not publicly available because of privacy and ethical restrictions, including patient confidentiality and Institutional Review Board requirements.

## Abbreviations

IEA: Inferior epigastric artery
TAE: Transcatheter arterial embolization
CT: Computed tomography
DSA: Digital subtraction angiography
DIC: Disseminated intravascular coagulation
SBP: Systolic blood pressure
RBC: Red blood cell
IQR: Interquartile range
GS: Gelatin sponge
NBCA: N-butyl-2-cyanoacrylate
PT-INR: Prothrombin time–international normalized ratio
OR: Odds ratio
aOR: Adjusted odds ratio
CI: Confidence interval
IRB: Institutional review board
