# Supplementary material for: Collateral-pathway–oriented clinico-angiographic framework for inferior epigastric artery–related hemorrhage: embolization planning and clinical outcomes
Source: CVIR Endovasc. 2026 Jul 29;9:91. doi: 10.1186/s42155-026-00742-3 (PMC13415429; doi:10.1186/s42155-026-00742-3)
Supplement: Supplementary file 1 — Supplementary Material 1: Table S3a. Sensitivity analysis: clinical etiology/setting substituted for the clinico-angiographic category in logistic regression for clinical failure. Notes: Sensitivity analysis: logistic regression using clinical etiology/setting in place of the clinico-angiographic category. Regression analysis set: Type D excluded (n = 59). * Lactate and RBC transfusion were excluded from the multivariable model due to collinearity with SBP and DIC. † Multiple trauma was excluded from the multivariable model because it occurred exclusively within pelvic fracture cases (perfect confounding). Table S3b, S3c. Sensitivity analyses using hemostasis-focused endpoints (IEA-territory rebleeding and non-death hemostatic failure) to address potential confounding by all-cause mortality in polytrauma. Table S3b. Outcome = IEA-territory rebleeding (CT/angiography-confirmed), Type D excluded (n = 59). Notes: Outcome events: 3/59, all in Type C (consistent with Table 2). Firth penalized logistic regression was used due to sparse events and quasi-separation. Table S3c. Outcome = Hemostasis-focused failure (IEA-territory rebleeding OR additional hemostatic intervention), excluding death-only cases without documented IEA-territory rebleeding/intervention, Type D excluded (n = 59). [file 42155_2026_742_MOESM1_ESM.docx]

**Supplementary Table S3a. Sensitivity analysis: clinical etiology/setting substituted for the clinico-angiographic category in logistic regression for clinical failure**

| **Variable** | **Univariable OR (95% CI)** | **p value** | **Multivariable aOR (95% CI)** | **p value (multivariable)** |
| --- | --- | --- | --- | --- |
| Etiology/setting: Pelvic fracture vs Abdominal wall | 16.5 (3.8–72.0) | <0.001 | 4.3 (1.2–15.5) | 0.03 |
| Etiology/setting: Postpartum vs Abdominal wall | 4.8 (0.9–26.0) | 0.08 | — | — |
| DIC present | 5.8 (1.7–19.2) | 0.004 | 3.5 (1.0–11.8) | 0.04 |
| SBP <80 mmHg before TAE | 5.0 (1.6–16.0) | 0.006 | 3.0 (1.0–9.2) | 0.049 |
| Lactate ≥4 mmol/L | 3.9 (1.2–12.8) | 0.02 | —* | —* |
| RBC transfusion ≥10 units (24 h before TAE) | 3.6 (1.1–12.2) | 0.03 | —* | —* |
| Multiple trauma present | 3.1 (0.9–11.0) | 0.09 | —† | —† |

**Notes:**

Sensitivity analysis: logistic regression using clinical etiology/setting in place of the clinico-angiographic category.

Regression analysis set: Type D excluded (n=59).

* Lactate and RBC transfusion were excluded from the multivariable model due to collinearity with SBP and DIC.

† Multiple trauma was excluded from the multivariable model because it occurred exclusively within pelvic fracture cases (perfect confounding).

**Supplementary Table S3b, S3c. Sensitivity analyses using hemostasis-focused endpoints (IEA-territory rebleeding and non-death hemostatic failure) to address potential confounding by all-cause mortality in polytrauma**

**Supplementary Table S3b Outcome = IEA-territory rebleeding (CT/angiography-confirmed), Type D excluded (n=59)**

| Variable | Firth OR (95% CI) | p value |
| --- | --- | --- |
| Type C vs Type A | 9.8 (1.6–>99) | 0.01 |
| Type B vs Type A | 0.8 (0.0–14.5) | 0.86 |
| DIC present | 2.4 (0.4–15.1) | 0.34 |
| SBP <80 mmHg | 2.1 (0.3–13.7) | 0.45 |

**Notes:**

**Outcome events: 3/59, all in Type C (consistent with Table 2).**

**Firth penalized logistic regression was used due to sparse events and quasi-separation.**

**Supplementary Table S3c Outcome = Hemostasis-focused failure (IEA-territory rebleeding OR additional hemostatic intervention), excluding death-only cases without documented IEA-territory rebleeding/intervention, Type D excluded (n=59)**

| Variable | Firth aOR (95% CI) | p value |
| --- | --- | --- |
| Type C vs Type A | 4.1 (0.9–19.8) | 0.07 |
| DIC present | 3.0 (0.8–12.1) | 0.10 |
| SBP <80 mmHg | 2.6 (0.7–10.2) | 0.15 |
